# Supplementary material for: The Effect of Disulfiram and N-Acetylcysteine, Potential Compensators for Sulfur Disorders, on Lipopolysaccharide-Induced Neuroinflammation Leading to Memory Impairment and the Metabolism of L-Cysteine Disturbance
Source: Molecules. 2025 Jan 27;30(3):578. doi: 10.3390/molecules30030578 (PMC11820383; doi:10.3390/molecules30030578)
Supplement: Supplementary file 1 [file molecules-30-00578-s001.zip › molecules-3299773-supplementary.pdf]

Supplementary part

Figure S1 shows photos of the objects used during NOR and OL tests.

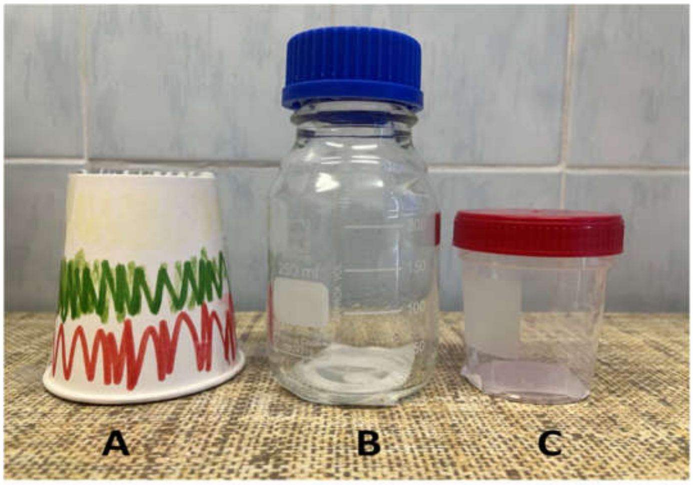

Fig. S1. Objects used in the NOR and OL tests. Object A - paper cup (painted with colors), object B - glass bottle with a blue cap, object C - plastic cup with a red cap.
